# Diagnostic clues of IOP pulsation on applanation tonometry in carotid-cavernous fistula patients

**DOI:** 10.1186/s12886-022-02254-9

**Published:** 2022-01-24

**Authors:** Hyunkyu Lee, Sumin Yoon, Sehyun Baek

**Affiliations:** 1 Department of Ophthalmology, Korea University College of Medicine, Ansan Hospital, Ansan, South Korea; 2 Department of Ophthalmology, Korea University College of Medicine, Guro Hospital, Seoul, South Korea

**Keywords:** Applanation tonometry, Carotid-cavernous fistula, Goldmann tonometry, IOP pulsation

## Abstract

**Background:**

Carotid-cavernous fistula (CCF) is an abnormal communication between the cavernous sinus and the carotid arterial system and exhibits typical symptoms of red eye, diplopia, blurred vision, headache, and murmur. However, the symptoms for CCF may vary and can lead to misdiagnosis. IOP pulsations provide a hint leading to suspicion of CCF. We report three cases related to CCF differential diagnosis: two cases of CCF patients and one case of conjunctivitis with corkscrew conjunctival vessels.

**Case presentation:**

The case 1 patient, with a typical unilateral CCF, exhibited significant IOP pulsation in Goldmann tonometry measurements in the affected eye. The case 2 patient did not show typical symptoms of CCF except asymmetric upper eyelid swelling (right > left). In clinical evaluation, IOP elevation in the right eye and IOP pulsation in both eyes were noted. Based on radiology, the patient was diagnosed with bilateral CCF. The case 3 patient was referred to our institution for differential diagnosis of CCF. The patient had corkscrew conjunctival vessels in both eyes, which had appeared after he had been revived through CPR (cardiopulmonary resuscitation) 25 years prior. IOP pulsation was not observed in Goldmann tonometry. Radiology test result for arterio-venous fistula was negative in the case 3 patient.

**Conclusion:**

For diagnosis of CCF, IOP pulsation by Goldmann applanation tonometry exhibits a good correlation with the disease in our cases and provides useful diagnostic clues.

**Supplementary Information:**

The online version contains supplementary material available at 10.1186/s12886-022-02254-9.

## Background

Carotid-cavernous fistula (CCF) is defined as an abnormal shunt from the carotid artery or its branches to the cavernous sinus. An abnormal shunt in CCF impedes normal venous drainage and causes symptoms such as red eye, diplopia, blurred vision, headache, and murmur [1]. Radiology tests including computed tomography and angiography (CT/A) and/or magnetic resonance imaging (MRI/A), ultrasound, and digital subtraction angiography (DSA) are good diagnostic tools for CCF. Those tests can detect an abnormal arterio-venous connection with high sensitivity as long as CCF is suspected [2]. However, misdiagnosis of CCF is not uncommon because of the varying symptoms of the disease. In fact, many authors have reported misdiagnosed CCF cases [3–5].

Through a fistula, high-pressure pulsating waves from the artery propagate into the vein. Based on the pathology of the disease, we assume that patients with CCF may exhibit intraocular pressure (IOP) pulsations in measurement with applanation tonometry according to pulsating arterial blood, and elevated IOP in CCF patients has been well noted. However, IOP pulsation evaluation with Goldmann tonometry for clinical diagnosis of CCF is uncommon. We believe that IOP pulsations can provide hints leading to suspicion of CCF. Therefore, we introduce three CCF cases related to differential diagnosis based on IOP pulsation signs in Goldmann tonometry. The study was approved by the Institutional Review Board of Korea University Hospital.

## Clinical presentation

### Case 1) unilateral CCF in the right eye

**Fig. 1:**
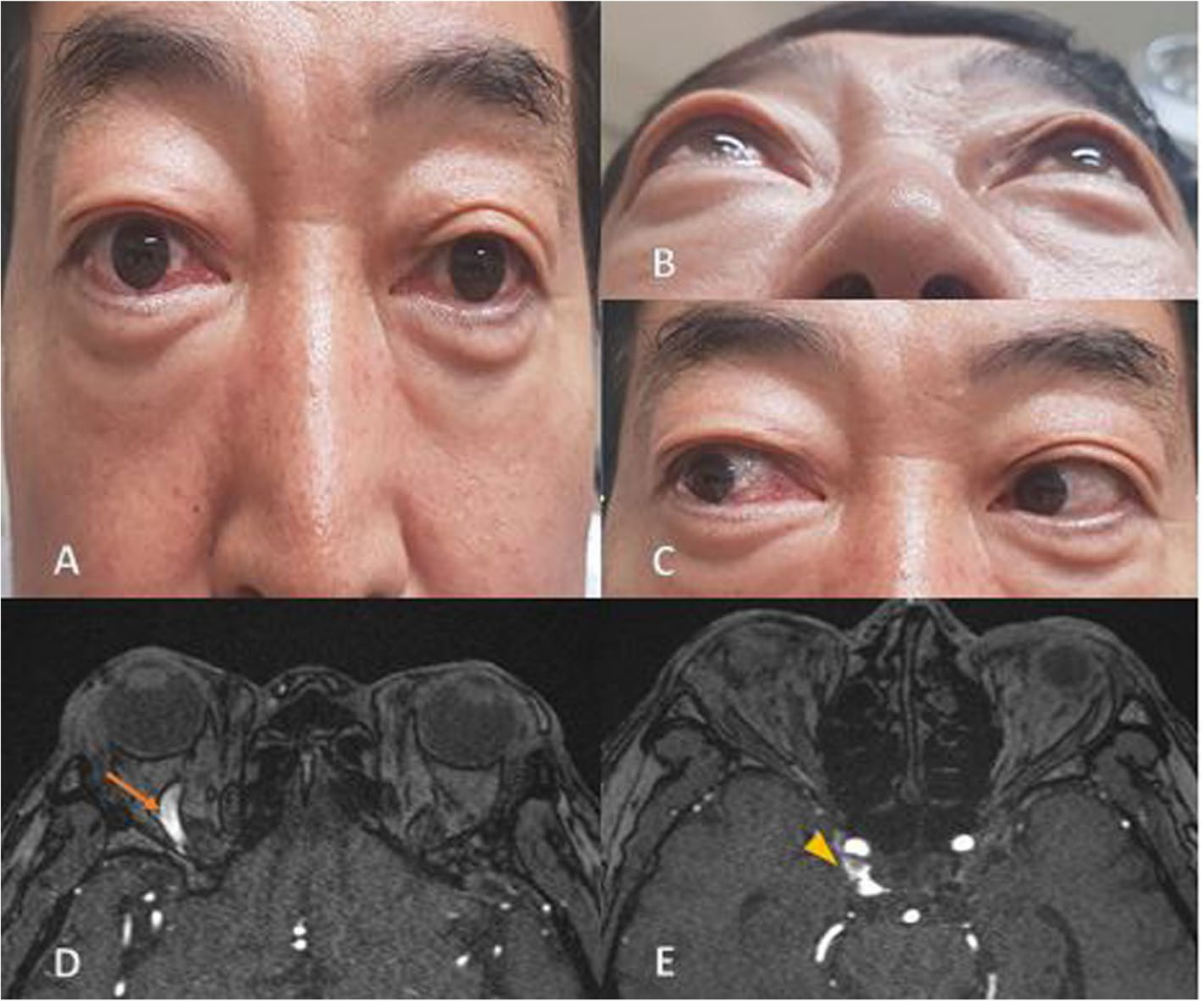
Photographs of the case 1 patient and his MRA. **A**, **B** The patient has proptosis and conjunctival injection in the right eye. **C** Conjunctival corkscrew vessels and chemosis as shown by lateral gaze limitation. **D** Superior ophthalmic vein (SOV) enlargement of the right eye (Arrow). **E** Shunt from right internal carotid artery (ICA) (Arrow head)

A 64-year-old male complained of diplopia beginning 1 week prior. Conjunctival injection and prominent proptosis were noted in his right eye (Fig. 1a, b). The patient reported no murmur symptom. Visual acuity was 1.0/1.0. Slit-lamp examination revealed a clear cornea and a quiet anterior chamber. Lateral gaze limitation on the right eye was noted in the extraocular motility test (Fig. 1c). IOP was 22 and 11 mmH by Goldmann tonometry in the right and left eyes, respectively. Also, definite IOP pulsations were observed only in the right eye (Supplemental digital content 1, Video 1). The patient presented with typical symptoms of CCF and showed definite IOP pulsations in Goldmann tonometry measurements of his affected eye. MR angiography was performed and showed an engorged right superior ophthalmic vein and an enhancing right cavernous sinus. Finally, the patient was diagnosed with indirect CCF with sixth cranial nerve palsy.

### Case 2) bilateral CCF

**Fig. 2:**
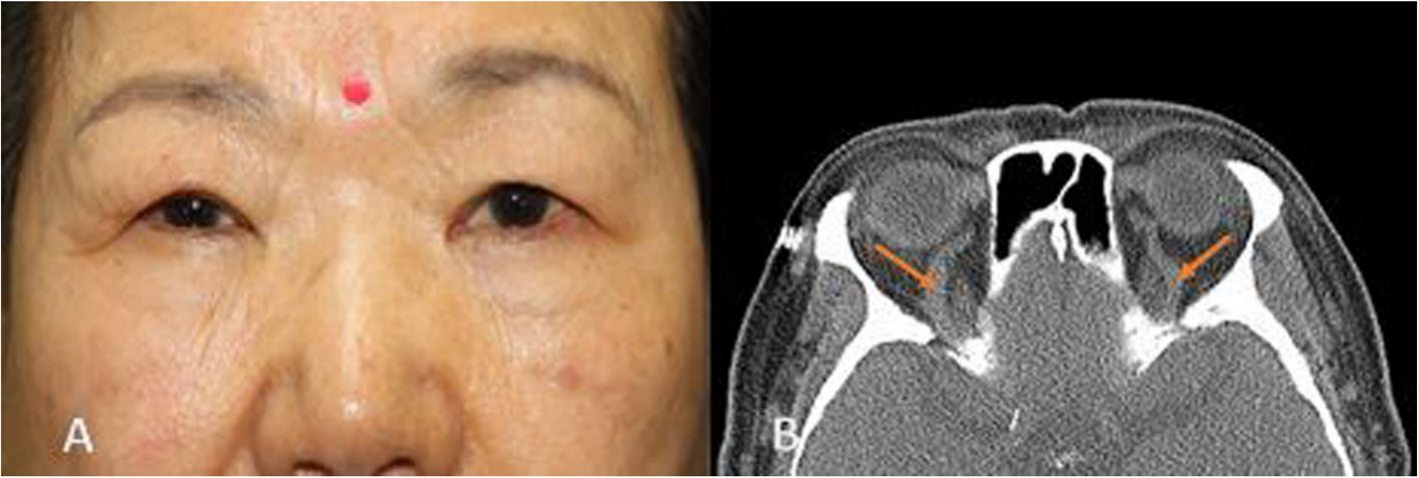
Photographs and CT image of the case 2 patient. **A** Asymmetric eyelid swellings (right > left) were noted. **B** Orbital CT (transverse view) revealed bilateral SOV enlargement (Arrows)

A 77-year-old woman reported upper eyelid swelling starting approximately 1 year previous. Asymmetric swelling on the upper eyelids was noted: moderate on the right, mild on the left (Fig. 2). There were no identifiable abnormal findings on slit-lamp examination. IOP of the right eye was 5 mmHg higher than that of the left eye but still within the normal range (Right eye: 20, Left eye: 15 mmHg). IOP pulsation was observed in both eyes (Supplemental digital content, Video 2a, b). Based on findings of IOP asymmetry and IOP pulsation with Goldmann tonometry, CCF was suspected. Additional history was gathered and revealed that the patient experienced murmur symptom when lying down at night. On CT scan, bilateral superior ophthalmic vein enlargement was observed, and the patient was diagnosed with Barrow type C CCF. Superior and inferior retinal nerve fiber layer (RNFL) defects were observed in the right eye on optical coherence tomography (OCT). The patient was treated with Xalost-S eye drops (0.005% latanoprost, Taejoon Pharm Co., Ltd.) to the right eye and underwent coil embolization.

### Case 3) conjunctivitis with corkscrew conjunctival vessels

**Fig. 3:**
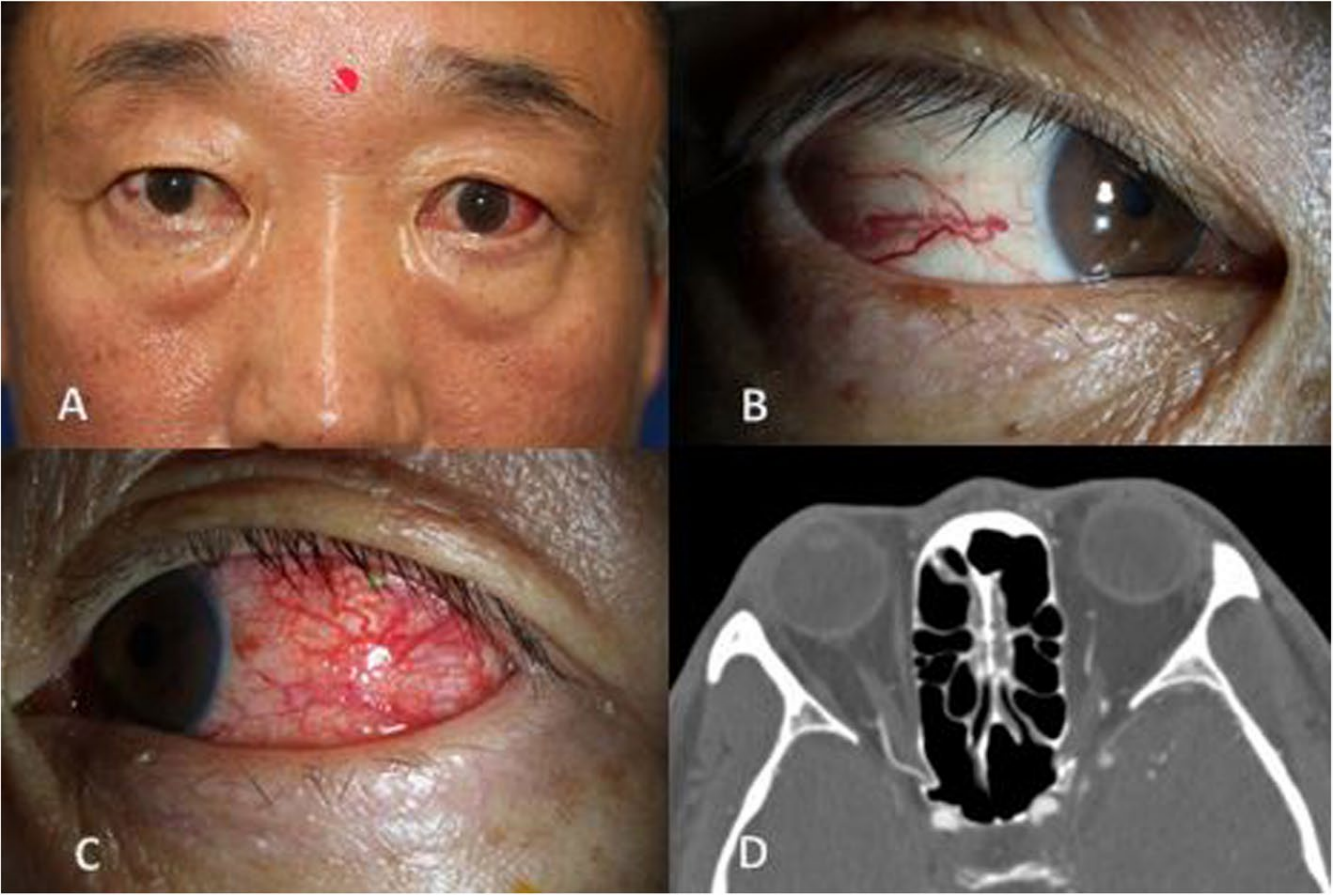
Photographs and CTA image of the case 3 patient. **A** The patient presented with conjunctival injection of his left eye. **B**, **C** Engorged corkscrew conjunctival vessels in both eyes. **D** CT angiography reveals no enlargement of the SOV

A 55-year-old male was referred to our institution for differential diagnosis of CCF. The patient reported that conjunctival redness of his left eye started 3 days prior. In slit exam, the patient exhibited bulbar conjunctiva injection on the temporal side of the left eye and corkscrew conjunctival vessels on both eyes (Fig. 3), which had appeared 25 years previous after cardiopulmonary resuscitation (CPR). The patient exhibited the most common sign of CCF, corkscrew conjunctival vessels, but it was chronic, and no other identifiable signs of CCF such as IOP pulsation, IOP elevation, proptosis, chemosis, extraocular muscle limitation, and optic neuropathy were noted (Supplemental digital content, Video 3a, b). Further radiology evaluations with CT angiography revealed no remarkable findings for CCF. Although negative CT results could not exclude CCF, the diagnosis of CCF was unlikely. The patient was treated with Lotepro eye drops (Loteprednol etabonate 0.5%, Incepta Pharmaceuticals Ltd.) to the left eye for conjunctival injection. The symptom improved after 2 weeks.

## Discussion and conclusion

CCF is defined as abnormal shunt from the carotid artery or its branches to the cavernous sinus. CCF can be classified as direct or indirect depending on whether it is supplied by the carotid artery or its branches. Ophthalmological symptoms and signs such as diplopia, headache, proptosis, ophthalmoplegia, chemosis, episcleral corkscrew vessels, IOP elevation, and blurred vision suggest CCF. However, CCF can exhibit a heterogeneous presentation that can be misinterpreted or missed if there is not a high index of suspicion [5]. In fact, there have been many case reports of CCF misdiagnosed as other diseases such as conjunctivitis and myasthenia [3–5]. Once CCF is suspected, it can be effectively evaluated through imaging, indicating the importance of clinical suspicion. Positive IOP pulsation in Goldmann tonometry provides a diagnostic clue in clinical evaluations by provoking suspicion of CCF and serving as a bridge for further imaging tests.

We presented three cases related to differential diagnosis of CCF. IOP pulsation on Goldmann tonometry exhibited good concordance with CCF diagnosis. The case 1 patient presented with typical signs of CCF of conjunctival injection, proptosis, IOP asymmetry, and abducent nerve palsy, and IOP pulsation supported this. In case 2, the patient exhibited only eyelid swellings on both eyes, suggesting thyroid eye disease, allergies, or inflammatory diseases that are more likely than CCF. Based on findings of asymmetric IOP and bilateral IOP pulsation, further history was collected to reveal that the patient experienced murmur while lying down at night. CCF patents often do not report their murmurs before being asked because they do not associate abnormal auditory symptoms with ocular problems. The case 3 patient had corkscrew conjunctival vessels in both eyes. Although corkscrew vessels are a pathognomonic finding of CCF, as onset was chronic for this patient and IOP pulsation was negative, the patient was determined to not have CCF.

There are several questions on the topic of IOP pulsation including presence in healthy subjects on Goldmann tonometry and amplitude of IOP pulsation as a reference value for diagnosis of CCF. There have been few studies on this topic. C Kaufmann et al. measured IOP pulsation in 150 healthy subjects with dynamic continuous tonometry and found that pulse amplitude readings ranged from 0.9 to 7.2 mmHg (median, 3.0 mmHg) [6]. Golnik and Miller reported that the pulsating amplitude between maximum and minimum IOP measured by a pneumotonometer was higher in CCF patients [7]. Goldmann tonometry cannot show IOP pulsation with digits for objective comparison like other tonometers, but it is available in more clinics, allowing doctors to check for presence/absence and degree of pulsation in a semi-quantitative manner. In this study, CCF patients had a difference in splitting as the semicircle dropped sharply when measuring IOP. However, due to the small number of patients, detailed objectification such as quantification is difficult. In order to quantify and compare the difference in IOP pulsation between normal people and CCF patients, further studies on IOP pulsation with Goldmann tonometer in a larger number of CCF patients and with a control group would clarify its clinical meaning and value in CCF.

In conclusion, arterial blood flow in CCF can cause pulsation of IOP in applanation tonometry. IOP pulsation can provide a diagnostic clue in clinical evaluation of CCF patients by provoking suspicion of the vascular disease. Further studies are required to elucidate the diagnostic importance and reference value of IOP pulsation in CCF.

## Supplementary Information

**Additional file 1: Video 1.** IOP pulsation in Goldmann applanation tonometry in case 1 patient. Applanation tonometry rings viewed through the Goldmann prism swings and shows definite IOP pulsation in right eye of the patient.

**Additional file 2: Video 2.** IOP pulsation in Goldmann applanation tonometry in case 2 patient. (A, B) There are signs of IOP pulsation in both eyes.

**Additional file 3: Video 3.** IOP pulsation in Goldmann applanation tonometry in case 3 patient. (A, B) Fluorescein semi-circles in the prism head are seen to meet and form a stable horizontal ‘S’ shape. There are no IOP pulsation signs in both eyes

## Data Availability

The datasets used and/or analysed during the current study are available from the corresponding author on reasonable request.

## Abbreviations

CCF: Carotid-cavernous fistula
CT/A: Computed tomography and angiography
MRI/A: Magnetic resonance imaging
DSA: Digital subtraction angiography
IOP: Intraocular pressure
RNFL: Retinal nerve fiber layer
OCT: Optical coherence tomography
CPR: Cardiopulmonary resuscitation
